# Measuring Treatment Response in Progressive Multiple Sclerosis—Considerations for Adapting to an Era of Multiple Treatment Options

**DOI:** 10.3390/biom11091342

**Published:** 2021-09-10

**Authors:** Nik Krajnc, Thomas Berger, Gabriel Bsteh

**Affiliations:** 1 Department of Neurology, Medical University of Vienna, 1090 Vienna, Austria; nik.krajnc@meduniwien.ac.at (N.K.); thomas.berger@medunwien.ac.at (T.B.); 2 Department of Neurology, University Medical Centre Ljubljana, 1000 Ljubljana, Slovenia

**Keywords:** multiple sclerosis, treatment response, clinical trial, outcome measure, disability progression

## Abstract

Disability in multiple sclerosis accrues predominantly in the progressive forms of the disease. While disease-modifying treatment of relapsing MS has drastically evolved over the last quarter-century, the development of efficient drugs for preventing or at least delaying disability in progressive MS has proven more challenging. In that way, many drugs (especially disease-modifying treatments) have been researched in the aspect of delaying disability progression in patients with a progressive course of the disease. While there are some disease-modifying treatments approved for progressive multiple sclerosis, their effect is moderate and limited mostly to patients with clinical and/or radiological signs of disease activity. Several phase III trials have used different primary outcomes with different time frames to define disease progression and to evaluate the efficacy of a disease-modifying treatment. The lack of sufficiently sensitive outcome measures could be a possible explanation for the negative clinical trials in progressive multiple sclerosis. On the other hand, even with a potential outcome measure that would be sensitive enough to determine disease progression and, thus, the efficacy or failure of a disease-modifying treatment, the question of clinical relevance remains unanswered. In this systematic review, we analyzed outcome measures and definitions of disease progression in phase III clinical trials in primary and secondary progressive multiple sclerosis. We discuss advantages and disadvantages of clinical and paraclinical outcome measures aiming for practical ways of combining them to detect disability progression more sensitively both in future clinical trials and current clinical routine.

## 1. Introduction

Multiple sclerosis (MS) is a chronic immune-mediated disease of the central nervous system, pathophysiologically characterized by inflammation, as well as neurodegeneration occurring in even early stages of the disease [1]. While 10–15% of patients display a progressive disease course from onset, called primary progressive MS (PPMS), natural history studies have indicated that about two-thirds of untreated patients with an initially relapsing course develop a progressive disease course, called secondary progressive MS (SPMS), after a mean of 15–20 years with broad interindividual variance [2,3,4]. Although advances in the treatment of relapsing MS have resulted in both a decrease and a delay of progressive MS (PMS), the degree of disability accrual is defined by PMS and the conversion to SPMS remains the single most important predictor of long-term outcome [5,6,7,8,9,10].

Encouragingly, efforts in recent years have yielded the first effective treatment options for PMS, albeit currently not as effective as in RMS [11,12]. Both the continuing quest for finding effective treatments for PMS and the growing challenge for navigating and sequencing treatment options require reliable methods for assessing response or failure of a given treatment. 

As we currently still lack treatments with the potential to repair or restore neuroaxonal damage, the basic principle of treatment response remains as prevention of acute or chronic inflammation-driven deterioration, i.e., disease or disability progression dependent or independent from clinical relapses. To establish the prevention of deterioration in a valid way requires testing instruments sensitive enough to detect deterioration but also specific and reliable enough to distinguish true deterioration from performance fluctuations and measurement-inherent errors. Equally important, outcome measures need to be able to answer whether the detected changes are clinically relevant, both at the moment of assessment and in terms of predicting future changes. 

Anticipating the availability of multiple treatment options for PMS in the not-too-distant future, outcome measures will also be required for evidence-based decision making to determine when to change or add DMT in PMS. In this regard, it is also to be defined what is the minimal or maximal time frame required to measure the efficacy or failure of DMT. 

Both successful and unsuccessful phase III clinical trials using different outcome measures with different time frames to define disease progression when evaluating the efficacy of DMT agents in PMS provide instructive data to compare and assess outcome measures. In that way, we analyzed the phase III clinical trials dealing with PMS with a special focus on the applied outcome measures and the definition of disability progression. Further on, we open the question of the frequency of patients’ evaluation in order to confirm the progression’s sustainability. Finally, we present our view on what should be taken into account when measuring DMT response both when planning future trials and treating patients with PMS.

## 2. Methods

### 2.1. Search Methods

A review of the literature concerning phase III clinical trials in PMS was performed using PubMed. Search terms included the following: progressive, multiple sclerosis, disease-modifying treatment AND phase III trial. The last search was performed on the 30th of June 2021. This search yielded 63 results.

### 2.2. Selection Criteria

**Figure 1:**
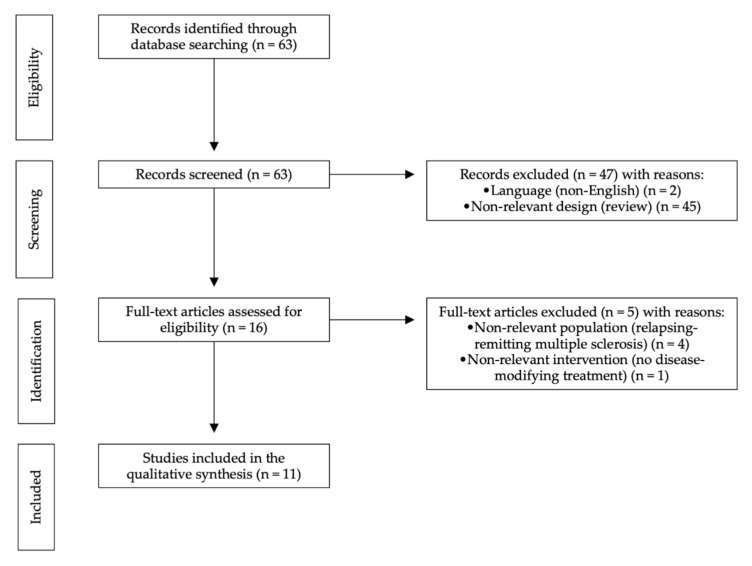
PRISMA flow diagram demonstrating included and excluded papers, and the reasons for exclusion in this systematic review.

Our selection criteria were language (English) and article type (clinical trial). After selection criteria were applied, 48 articles were excluded. The results were hand-searched to exclude additional nonrelevant publications. We have chosen to include only clinical trials published after 1993. The systematic review was prepared according to the latest PRISMA (Preferred Reporting Items for Systematic Reviews and Meta-Analyses) guidelines (see Figure 1).

## 3. Results

We identified 12 outcome measures from 11 trials dealing with the efficacy of DMT in both PPMS and SPMS. We present their characteristics and designs in Table 1.

### 3.1. Expanded Disability Status Scale

The Expanded Disability Status Scale (EDSS) is the mainstay primary outcome measure of disability progression in MS. Developed in 1983, it is widely used in both studies and clinical routine and is, thus, widely familiar to MS clinicians [13]. Based on a standard neurological examination, MS-related impairment is quantified on a 0–10 scale emphasizing walking ability (EDSS score 4.0–6.0), or other functional impairments (EDSS score ≥ 6.5) [13].

There is some variability in the definition of disability progression: ORATORIO, OLYMPUS, and SPECTRIMS adopted the now-widely recommended definition of an increase of ≥1.0 points from baseline scores ≤5.5 or ≥0.5 points (EDSS ≥ 6.0), whereas the PROMiSE and EXPAND trials used the EDSS ≥ 5.5 as the threshold for a 0.5-point step change [11,12,14,15,16]. As EDSS 6.0 defines the need for an assistive device, it seems to be a more reasonable cut-off value for a 0.5-point change to detect disability progression. The interferon β 1b trial defined disability progression as an increase of ≥1.0 point regardless of the baseline EDSS, but the trial only enrolled patients with a lower level of disability (EDSS 1.0–3.5) [17].

The minimum time period of sustainment required in order to meet the criteria for disability progression also varies considerably from 12 weeks (ORATORIO and OLYMPUS), to 3 months (INFORMS, PROMiSE, and EXPAND) or 6 months (interferon β 1b study) [11,12,14,16,17,18]. Of note, worsening needs to be confirmed in the same functional score in which it was detected at baseline in order to maintain the validity of the EDSS [19]. In that way, the necessary time frame to determine disease progression with EDSS can be inferred as ≥3 months. 

In the MIMS study, however, only the change in EDSS between baseline and month 24 was evaluated [20]. The use of a roving EDSS reference value, in which the increase or decrease in EDSS has to be separated from the last EDSS assessment by at least 24 or 48 weeks, allows the more sensitive measurement of disability progression in comparison to the conventional fixed study baseline EDSS but decreases specificity [21,22].

Although EDSS is easy to administer, well-known, and includes most functional systems that might be affected in MS, it also has some limitations [23]. Due to its nonlinear nature, an increase of 1.0 point has a different significance for each level [24,25]. EDSS also lacks sensitivity for detecting a change in clinical status in patients with a progressive course, especially upper-extremity and cognitive dysfunction, as it is heavily weighted toward ambulation at higher degrees of disability [26,27,28,29]. Moreover, it also lacks inter- and intra-rater reliability, the latter being more variable with lower EDSS scores [30,31,32,33].

### 3.2. Timed 25-Foot Walk Test

The Timed 25-Foot Walk Test (T25FW) evaluates the time required to walk the distance of 25 feet (7.62 m) in the patient’s usual manner, including potential use of assistive devices [34]. The test is performed twice, and the average of both trials is scored. T25FW is reliable over short and long periods of time, has a high degree of validity, and responds to a clinically meaningful change in disability progression [34,35,36,37,38].

The INFORMS and ASCEND clinical trials used the T25FW as one of the primary outcome measures [18,39]. An increase of ≥20% in the T25FW was defined as clinically significant [35,40,41,42,43,44]. The necessary time frame to determine disease progression with T25FW is ≥3 months.

T25FW has advantages as it is more sensitive to detect disease progression compared to EDSS, can be easily administered, and is inexpensive and applicable among a wide range of patients with different disability levels [45,46]. However, with patients reaching wheelchair dependence (EDSS ≥ 7.0), it becomes less useful in determining disease progression (ceiling effect), and it also shows some flooring effects, which, however, seems to be less important in patients with PMS [47].

### 3.3. 9-Hole Peg Test

The 9-Hole Peg Test (9HPT) evaluates manual dexterity by measuring the time in which a patient moves 9 pegs one by one into 9 holes on a board and back into the box [48]. The test is performed twice with each hand, and the time is averaged for each side separately [49]. An alternative scoring with the number of pegs placed in 50 or 100 s can be recorded; in this case, results are expressed as the number of pegs placed per second [50,51].

The INFORMS and ASCEND clinical trials used the 9HPT as one of the primary outcome measures [18,39]. An increase of ≥20% in the 9HPT was defined as clinically significant as described before [42,43]. However, the minimum 9HPT score at baseline should be at least 21 s in order to detect such a change [52]. The necessary time frame to determine disease progression with 9HPT is ≥3 months.

The advantages of 9HPT are that it is easily accessible, requires no training, and evaluates measures that are not included in EDSS. Besides, it shows excellent test–retest reliability, as well as intra- and interrater reliability [53]. However, 9HPT demonstrates a practice effect with each repetition, as well as floor and ceiling effect [52]. The former can be avoided with the calculation of pegs per second in people with severe upper limb dysfunction [54].

### 3.4. Paced Auditory Serial Addition Test

The Paced Auditory Serial Addition Test (PASAT) has been used since 1974 as a common research tool for different neurological disorders, including MS [55]. It assesses the auditory information processing capacity by the presentation of 60 numbers every 3 s to the patient who is asked to add each new digit to the one immediately prior to it [56]. The scores most commonly reported for the PASAT are the number of correct responses for each trial (maximum 60 points) or the total number of correct responses over all trials [55].

Only the IMPACT study used PASAT as one of the primary outcome measures, where a decrease of ≥20% in the PASAT score was defined as clinically significant [57]. The necessary time to determine disease progression with PASAT is at least 3 months.

PASAT has some advantages as it evaluates measures not included in EDSS and shows a high level of test–retest reliability [55]. However, it demonstrates a practice effect, which seems to be independent of the duration of the test–retest interval. Besides, it is perceived as a relatively difficult task even for individuals with high intellectual capability and can be interpreted differently (number of correct responses, the average correct response time, etc.) [58].

### 3.5. Symbol Digit Modalities Test

The Symbol Digit Modalities Test (SDMT) is a sentinel test for cognitive impairment in patients with MS (60% specificity and 91% sensitivity) [59,60]. Using a reference key, the test taker has 90 s to pair specific numbers with given geometric figures. The task evaluates sustained attention, capacity of concentration, and visuomotor speed [61,62,63].

SDMT was used in the EXPAND clinical trial in which a ≥4-point or ≥10% change in SDMT was considered clinically relevant [64,65]. The necessary time frame to determine disease progression with SDMT is ≥3 months.

SDMT is time-efficient, easy to administer, change-sensitive, and independent of language. It also correlates less strongly with EDSS and the other performance measures (T25FW and 9HPT), providing additional information by assessing functions not captured by the other measures [47]. It shows superior reliability, sensitivity, and greater patient acceptability compared to PASAT and, hence, SDMT is recommended for use instead of PASAT [64,65,66]. However, its normative values are affected by several factors such as age, sex, education, and cultural background [67]. Besides, it might demonstrate a slight practice effect, which is why a change in the key sequence is recommended for each repetition [68].

### 3.6. Low-Contrast Letter Acuity

The Low-Contract Letter Acuity (LCLA) chart is based on Early Treatment Diabetic Retinopathy Study High-Contrast Letter Acuity (HCLA) charts, but uses grey letters with 2.5% and 1.25% contrast levels as opposed to black letters (100% contrast level) [69]. Patients are asked to read letters from a 2 m distance proceeding top to bottom and from left to right. The score for each chart is quantified as the number of letters identified correctly with a maximum score of 70 letters [70]. LCLA shows good structure-function-correlation both with retinal atrophy and lesions in the posterior visual pathway [71,72,73].

The LCLA chart was first used as an exploratory outcome measure in the IMPACT clinical trial, where the clinically meaningful change was determined at a ≥7 letters loss, which is beyond the threshold of test–retest variability [74,75,76,77]. The necessary time frame to determine disease progression with the LCLA chart seems to be ≥3 months.

LCLA is easy to administer and interpret, provides a linear scale for statistical analysis, and shows excellent interrater reliability [70]. It correlates less strongly with EDSS and other performance measures, supporting its additive value to assess functions that are not captured by the latter [47]. Still, some correlation between LCLA and cognitive performance, measured by SDMT, has been described lately [78]. LCLA charts have some limitations such as a potential floor effect with lower contrast levels and a ceiling effect with higher contrast levels as most letters on the chart are easily readable with no potential for improvement over time [79]. Besides, the conditions in which the test is performed should be well-controlled (no surface reflections on the charts, even backlighting in a retro-illuminated cabinet) in order to receive comparable data.

### 3.7. Scripps Neurologic Rating Scale

The Scripps Neurologic Rating Scale (SNRS) is a summary measure of individual components comprising a neurological examination, designed for use in MS [80]. The assignment of points in the SNRS reflects the examiner’s clinical assessment of each component in the neurological examination; points are then summed to arrive at the overall SNRS score. The responsiveness of most of the components seems to be in the small to moderate range; however, some components, such as the bladder, bowel, or sexual dysfunction, are insensitive to change [81]. Still, SNRS may be more sensitive than EDSS alone.

Only the Cladribine Study included SNRS as one of two primary outcome measures (besides EDSS) [82]. Although a change in SNRS was noted every month, additional analysis showed that a decrease in SNRS for ≥10 points after one year was meaningful in the sense of progression [82]. In that way, the necessary time frame to determine disease progression with SNRS seems to be ≥12 months.

Although SNRS seems to be more sensitive than EDSS, it is seldomly used and may not bring much additional value when determining disease progression. Besides, it seems that the necessary time frame to determine disease progression is considerably longer than in other measures.

### 3.8. Magnetic Resonance Imaging

In all mentioned clinical trials, imaging outcome measures have been used as secondary outcome measures, mostly the number of new/enlarging and/or gadolinium-enhancing lesions, with only a few of them including brain volume change as well. Grey matter atrophy occurs in all phenotypes of MS and is associated with disability accumulation [83]. Recently, cut-offs to distinguish pathological brain atrophy related to MS from the physiological change have been established, with 0.40% per year performing best for detecting physical disability progression (65% sensitivity and 80% specificity) [84]. Cortical atrophy seems to accelerate in PMS compared to relapsing-remitting MS (−0.87% vs. −0.48%, respectively) [85]. Although measurement techniques allow good reproducibility and sensitivity to changes, there are several limitations that need to be taken into account (e.g., technical limitations and dependence on confounding factors) [86,87,88,89,90]. Besides, patients treated with DMT have a slight decrease in the brain volume in the first 6–12 months (pseudoatrophy) due to the resolution of inflammation and its associated edema [91]. A possible solution would be to perform a re-baseline MRI 3–6 months after DMT initiation, although this would lengthen the time of observation, as a necessary time frame between two MRIs seems to be ≥1 year.

On the other hand, none of the clinical trials used spinal cord atrophy as an outcome measure, although it is considered a major driver of disability accumulation and is increasingly recognized as an additional imaging outcome measure besides brain atrophy [92]. The measurement of the cross-sectional area of the spinal cord presents a sensitive biomarker, especially as the estimated annual rate of spinal cord atrophy is greater when compared to the rate of brain atrophy in patients with MS (−1.78% vs. −0.5%) [93,94]. However, it remains to be elucidated what constitutes a relevant change in spinal cord atrophy as opposed to background noise or natural change. There are, however, some limitations that need to be considered. The spinal cord is a relatively small structure, which makes it difficult to obtain high reproducibility and responsiveness to changes, especially in a multi-center setting with variability in imaging protocols and scanners. As for brain MRI, a necessary time frame to determine disease progression is ≥1 year.

### 3.9. Composite Outcome Measures

The Multiple Sclerosis Functional Composite (MSFC) was developed in 1999 as an alternative approach to address the perceived shortcomings of the EDSS [48,95]. It contains three of the most important domains of the disease, including lower (T25FW) and upper extremity function (9HPT), and cognition (PASAT) [96]. To combine the results, Z-scores are calculated from each test and averaged to form a composite score [48].

The validity of MSFC as a clinical outcome measure is supported by several independent studies, including the IMPACT study. In this study, decreases in mean and median MSFC Z-scores were marked as progression [74]. The threshold for each of the measurements to detect clinically meaningful disability progression was reported at ≥20% [42,43,97]. The necessary time frame to determine disease progression with MSFC is ≥3 months.

MSFC is a more reliable tool than EDSS with the ability to be conducted quickly by a trained technician. Besides, the Z-score is a continuous and unitless variable, facilitating longitudinal analysis over time [98]. However, the use of Z-scores opens the question of its relation to the clinically meaningful change in function. The lack of a vision measure seems to be a weakness of the original MSFC, and its components may suffer from ceiling and floor effects depending on the level of disability. Still, MSFC seems to be more sensitive than EDSS in detecting disability progression [99].

As PASAT has not been shown to sensitively detect cognitive progression in PMS, a composite measure of disability progression, incorporating the EDSS, T25FW, and 9HPT, has been considered (EDSS-Plus) [37,97,100]. It should be differentiated from MSFC, which uses analytical methods to create a single score from different scales and is, in that way, difficult to be appropriately weighted, whereas a multicomponent endpoint combines different outcomes and analyzes them on their own.

The EDSS-Plus was used in the ASCEND and INFORMS clinical trial [18,39]. In both, disability progression was defined as an increase of ≥1.0 points (EDSS ≤ 5.5) or ≥0.5 points (EDSS ≥ 6.0), or an increase of ≥20% in the T25FW or 9HPT [18,39]. The necessary time frame to determine disease progression with EDSS-Plus is ≥3 months.

The EDSS-Plus is a more sensitive tool than EDSS alone, identifying 60% of patients as progressors, compared with 25%, 42%, and 34%, when using the individual tests alone (EDSS, T25FW, and 9HPT, respectively) [97]. This was confirmed in the INFORMS study, where the 3 month CDP for EDSS, T25FW, and 9HPT was 54%, 63%, and 34%, respectively, with a composite score confirming progression in 77% of patients [18]. Similar conclusions were drawn from the post hoc analysis of the ORATORIO clinical trial [101]. Apart from greater sensitivity, and assessing short-distance ambulation and upper-extremity function, both T25FW and 9HPT are continuous measures compared to EDSS, which has an ordinal scale.

Composite scores have several strengths by including the combination of different assessments of MS disease activity and describing treatment outcomes more comprehensively than its single parameters [102]. The use of composite endpoints broadens measures of the therapeutic effect and can decrease the required sample size or increase the trial’s ability to detect smaller efficacy levels [103]. It has to be kept in mind that the application of composite measures may increase the sensitivity to detect disability progression, but does not even out the intrinsic limitations of the single measures within the composite.

## 4. Discussion

We have identified several outcome measures dealing with different aspects of disease pathology, as well as with different definitions of and the sensibility to predict disease progression. Groups of patients involved in the clinical trials were relatively homogenous as mostly patients with EDSS 3.5–6.0 were included in the studies. Although the most commonly used primary outcome measure was EDSS, we have seen that others, especially composite outcome measures, show a higher level of sensitivity to detect disability progression.

Although the aim of this paper was not to address the definition of SPMS, it remains an important question that should not remain unanswered. Most of the clinical trials have used the definition of SPMS as previously diagnosed RRMS with signs of disability progression (EDSS change ≥ 1.0) before enrolment. In the previous years, a new definition of SPMS has been proposed with an increase of ≥1.0 points from baseline scores ≤5.5 or ≥0.5 points (EDSS ≥ 6.0) in the absence of a relapse, a minimum EDSS score of 4, and a pyramidal FS score of 2, confirmed over ≥3 months [104]. An extended discussion about its definition exceeds the aim of this paper but was recently published in a systematic review about the recognition of conversion to SPMS [105].

### 4.1. Detection of Disability Progression Depends on the Level of Disability

While natural history studies have suggested a relatively uniform pace of progression across patients, it is now well established that the velocity of disability progression—and thus the probability within a certain time frame—depends not only on the sensitivity of the measure but also on the level of disability at the starting point of a given observation period. Obviously, most data are available for EDSS in this regard, where the probability of disease progression significantly decreases for higher EDSS scores, particularly ≥7 [106,107].

Patients included in PMS clinical trials shared a comparable level of disability at scores 3.0–6.0; a question that should be put forward is how to evaluate progression in patients with a lower (EDSS ≤ 4.0) or higher (EDSS ≥ 7.0) level of disability. In those patient groups, the additional use of other measures to determine disability progression, such as 9HPT and SDMT, seems reasonable [108,109].

As the baseline measures appear to be predictive of future disability progression, the proposed outcome measure should be carefully chosen according to patients’ baseline data. Patients with a lower baseline T25FW (<6 s) show a lower rate of T25FW worsening; a cut-off of baseline T25FW ≥ 8 s predicts 40% of disability progression [45,110,111]. A cut-off of baseline 9HPT ≥ 21 s has been suggested, too, in order to detect a clinically meaningful change [52]. Moreover, patients with a lower baseline SDMT score (< 43) appear to have a higher chance for disability progression, measured by SDMT [64,66].

Among clinical outcome measures, the 9HPT could play an important role in those patients who maintain some degree of manual dexterity but are already wheelchair-dependent (EDSS ≥ 7.0). On the other hand, SDMT should be used in every patient regardless of the prior cognitive impairment or other level of disability, but it should be kept in mind that the sensitivity to change grows with SDMT lower than 43.

Moreover, with the reaching of higher degrees of disability, we should aim to take more paraclinical outcome measures into consideration. Lately, serum neurofilament light chain (sNfL) levels have been proven to correlate well with disability in both SPMS and PPMS patients and increase over time, even in those whose EDSS remained stable in the observation period [112].

### 4.2. The Absence of Worsening vs. Improvement of Disability

With potentially neuroprotective drugs being developed in the following years, it is important to distinguish between measuring the treatment effect in worsening vs. the absence of worsening as opposed to improvement vs. stability vs. worsening from a methodological point of view. In the current landscape of DMT for PMS, there is no treatment available achieving sustainable improvement. Thus, an ideal outcome measure would still only show the worsening of disability or stability over time, whereas the improvement of disability would be considered noise due to random variation or measurement error [113]. In that way, Koch et al. analyzed the reliability of the outcome measure and showed that the T25FW and the 9HPT have lower “improvement” rates compared to EDSS, consistent with less measurement error, suggesting them to be reliable outcomes [114]. Kalincik et al. suggested the comparison of the screening and baseline measurements in order to estimate their measurement errors, which could then inform definitions of disability progression and improvement for different baseline values [115].

Although disability improvement was not measured as a distinct outcome in most trials, there are some trials investigating potential neuroprotective drug effects aiming at improvement in outcome measures. A trial investigating high-dose biotin treatment in SPMS defined improvement as a decrease of ≥0.5 point (EDSS ≥ 6.0) or ≥1.0 point (EDSS ≤ 5.5) or a decrease of ≥20% for T25FW, and reported that 13% of patients in the biotin arm (compared to 0% treated with placebo) improved in disability [116]. However, according to a recent analysis, we could expect that approximately 15% of patients (14.6% in the ASCEND and 15.8% in the IMPACT study) would experience such an “improvement,” based only on the imprecision of the outcome measure [114]. Therefore, if trials are to use disability improvement as an outcome measure, the anticipated rates of the latter should be corrected accordingly. Importantly, the confounding influence of relapses and subsequent improvement have to be considered, which are often challenging to distinguish from progression, particularly in SPMS with superimposed relapses. To reduce this source of bias, definitions of disability progression should include sustainment for at least 6 months.

### 4.3. Time Frame for Objectivation of Treatment Response

Equally important to what defines treatment response is the time frame required to define treatment response, i.e., how long and how often do we need to measure. In most trials, disability worsening had to be confirmed after 3–6 months, and/or at the last trial visit in order to be labeled “sustained.” When comparing the results for 12 weeks and 24 weeks confirmed disease progression in the ORATORIO study, the magnitude of the effect of ocrelizumab on clinical endpoints was similar [11]. A recent analysis of Koch et al. showed that patients at the 3 month confirmed progression translated to sustained progression at 12 months in 66–88% of patients with SPMS [114,117]. However, the 3 to 6 month confirmed disability progression may still overestimate the accumulation of permanent disability by up to 30% [118]. In that way, disability progression, confirmed at the end of the study, seems to be more accurate, eliminating the effect of a potential relapse and truly determining progression independent of a relapse-associated disability, although possibly leading to fewer worsening events and increasing the required study sample size [119,120]. Hence, future clinical trials should focus more on relapse-independent disability progression, especially as there are some DMTs already available for clinically and/or radiologically active PMS, who are, however, lacking a sufficient effect on the prevention of disability progression. A recent study suggested a better responsiveness using a roving EDSS model, comparing the possible progression with the last EDSS before the progression instead of the study baseline. Whilst this increases the number of events and reduces the required sample size, it inevitably results in increased “false-positives” [22].

Clinical trials traditionally use observation periods of two years. In the SPECTRIMS study, the estimated probabilities of remaining progression free were calculated for each 3 month period throughout the study period. Treatment effects became visible after 9 months of treatment and were significant after 12 months, maintaining significance for each 3 month period throughout the remainder of the study [15]. In that way, the minimal necessary time frame to assess DMT response or failure seems to be at least one year. However, as disability progression remains a rare event, the observation period needs to be extended to at least two years in clinical trials even with a combination of multiple outcome measures. In clinical routine for assessment in individual patients, the required observation period will probably be considerably longer.

### 4.4. Other Outcome Measures

There are several other potential biomarkers of disease progression that have not been incorporated into phase III clinical trials yet. Optical coherence tomography (OCT) is a noninvasive tool that allows high-resolution measurement of retinal layer thickness, and has already been used in some phase 2 clinical trials in PMS. Peripapillary retinal nerve fiber layer (pRNFL) and ganglion cell-inner plexiform layer (GCIPL) thicknesses are robust indicators of neuroaxonal degeneration [121,122]. They are both significantly reduced in patients with MS regardless of prior optic neuritis, and are associated with both present and future physical and cognitive disabilities, as well as brain atrophy [121,122,123,124]. With a specificity of 90% and a sensitivity of 76%, an annual pRNFL thinning rate of more than 1.5 µm is able to distinguish between stable and progressive MS [125]. A recent study found that an annual loss in GCIPL above a cut-off ≥1 µm accurately identifies clinically progressing patients (87% sensitivity and 90% specificity) and presents an even stronger predictor of clinical progression than pRNFL does [126]. As it is a fast, noninvasive imaging method that reliably produces quantitative measures, it has great potential to test neuroprotective strategies over a short time frame (≥12 months).

Still, OCT has some limitations, too, especially in patients with PMS. With the progression of the retinal atrophy, at some point, the floor effect is reached, after which no more thinning is observable. However, the flooring effect is less prominent in GCIPL than in pRNFL [127]. Besides, OCT is not available in every center and requires a lot of expertise and data control in order to be valid.

sNfLs are an emerging biomarker garnering considerable interest over the past few years. NfLs are a major component of the neuronal cytoskeleton, and neuroaxonal damage causes their release into the extracellular space and further into the cerebrospinal fluid and the blood. NfL levels in the cerebrospinal fluid are associated with clinical and radiological activity and show good correlation with future worsening on the EDSS and brain and cervical spinal volume loss [128,129,130,131]. The advent of the Single Molecule Array (Simoa^®^) enabled highly sensitive quantitation of the NfL in the peripheral blood [128]. sNfL levels correlate with disability and increase over time, even in the absence of prior/subsequent disability progression, and are associated with various MRI parameters of neuroaxonal degeneration (T1 black holes, brain and spinal cord atrophy) [112,131,132,133]. sNfL can be easily obtained and should be therefore considered to be included as an outcome measure in newer progressive MS trials with a potential to detect disease progression more sensitively and especially in a shorter time frame (≥3 months).

sNfL still has some limitations that have to be considered. There are no established reference values that could be interpreted pathologic nor are they specific for MS, as they only imply neuroaxonal damage of any cause. Until now, no definition of an sNfL response to either treatment or disease progression has been established. Furthermore, only a limited set of data is available considering sNfL in patients with PMS [134].

### 4.5. Recommendations for Future Study Trials and Everyday Clinical Practice

Clinical trials in PMS require study populations with sufficient pre-test probability of disability progression in order to provide sufficient statistical power for detection of treatment effects and avoid type II error. For want of better prediction tools, this is accomplished by applying recent disability progression as an inclusion criterion. Currently, this is almost exclusively based on the assessment of progression by EDSS, which is insufficient, and we should strive to use more sensitive and complimentary measures. 

An important point concerns the sample size needed for each of the outcome measures in order to reliably and validly distinguish progressing and nonprogressing patients. This aspect has become particularly important with the occurrence of newly designed multi-arm trials for investigating DMT for PMS. Where EDSS was used as the primary outcome measure, approximately 20% of patients showed signs of progression within a period of two years. Based on this anticipated incidence in disease progression and an alpha level of 0.05 and power of 80%, at least 400 patients are required for a trial with 1:1 randomization. The same applies for other clinical measures, in which a 20% change is defined as clinically relevant (T25FW and 9HPT). For SDMT with a relevant change of 10%, the sample should be much larger (n = 870). While a combination of multiple clinical parameters would, in theory, reduce the sample size required to about 150 patients per treatment arm, more focus should be placed on biomarkers of neurodegenerative processes such as brain atrophy and retinal thinning. Paraclinical measures would allow us to reduce the sample size as, with higher sensitivity, the anticipated progression rate would be higher, too. Both brain atrophy and OCT measures (pRFNL and GCIPL) require a much smaller sample size in order to detect a clinically relevant change (n = 60 and 120, respectively) [135]. However, less is known about the relevant change in sNfL, and its levels are also dependent on the type of DMT [136].

In clinical trials in PMS, EDSS should be gradually replaced as a primary endpoint in determining disease progression by clinical composite scores such as EDSS-Plus in order to achieve greater sensitivity. Lately, a combinatorial weight-adjusted disability score (CombiWISE) has been developed, integrating four clinical scales, i.e., EDSS, SNRS, T25FW, and 9HPT [137]. However, these composite scores need to be formally validated as a surrogate marker of treatment response, i.e., long-term outcome, in prospective clinical trials. Clinical composite scores should be complemented as secondary or explorative outcome measures by paraclinical parameters, including cerebral or spinal MRI parameters, OCT markers, and sNfL with the goal of defining paraclinical patterns of subclinical disease progression associated with later clinical response. As MRI, OCT, and especially sNfL could determine the degree of neuroaxonal damage within a one-year interval, these patterns could then—after prospective validation—be used as surrogates enabling an earlier assessment of treatment response in order to both objectify and shorten the process.

However, clinical trials are still far away from the actual clinical routine, as not every clinical center has the capacity to perform all the suggested outcome measurements in order to sensitively detect disease progression in their patients. Patient-reported outcomes (PROs), which have also been used in several clinical trials, might seem a reasonable option to overcome a lack of both staff and equipment and might also reflect often neglected aspects such as patients’ quality of life and the ability to carry out activities of daily living. However, currently available patient-reported outcome measures are neither specific nor sensitive enough to determine disease progression to fulfil the required standards of reliability and objectivity [138].

**Figure 2:**
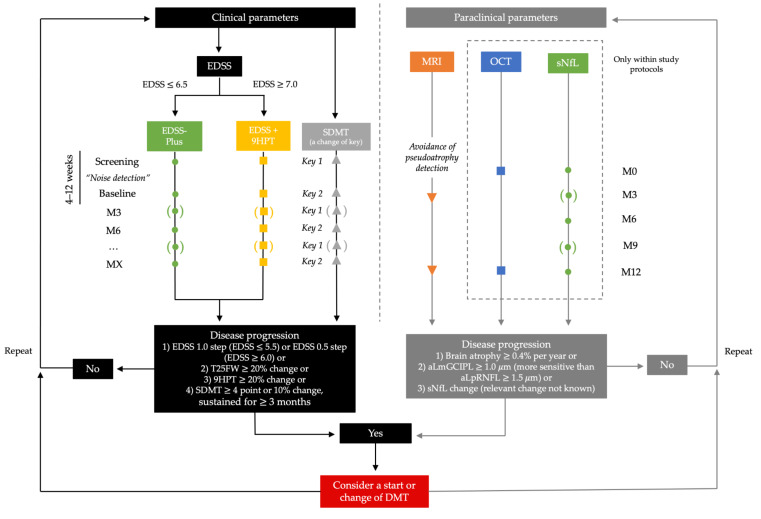
Proposed protocol for assessment of patients with progressive multiple sclerosis in clinical trials and everyday clinical routine. The measurements should be performed every 3 (clinical trials) or 6 months (everyday clinical routine) with 3 months desirable if possible. EDSS—Expanded Disability Status Scale, MRI—magnetic resonance imaging, OCT—optical coherence tomography, SDMT—Symbol Digit Modalities Test, sNfL—serum neurofilament light chain, T25FW—Times 25-Foot Walk Test, 9HPT—9-Hole Peg Test.

We would strongly advocate to at least adopt a minimal set of assessments of PMS patients in addition to EDSS. In ambulatory patients, the performance of T25FW and SDMT would significantly increase sensitivity with an additional capacity of 3–5 min required. In nonambulatory patients, 9HPT should be used instead of T25FW. Assessments should at least be conducted at an interval of six months, with three months desirable if possible (see Figure 2).

Regarding paraclinical assessments, brain and cervical spine MRI should be conducted at an interval of 1–2 years in order to detect signs of inflammatory disease activity and—where feasible—measure atrophy over time. Apart from that, we see a great potential in the bringing of the more available and reproducible biomarkers of disease progression, such as OCT and sNfL, into everyday clinical practice. However, while these cut-offs show good reliability on a group level, they are less reliable when assessing individual patients in everyday clinical practice. That should be taken into account when determining progression independently of study protocols, and needs to be further elucidated.

Anticipating the availability of multiple treatment options for PMS in the not-too-distant future, clinical outcome measures will have to provide the basis for the determination of treatment response or failure and, consequently, when to change DMT in PMS. Considering efficacy profiles of currently available DMTs, as well as substances in the pipeline, the prevention of further disease progression rather than sustainable improvement will represent a realistic treatment goal for the foreseeable future. However, we should not be content with establishing a stable EDSS, but rather strive toward adopting a multivariate definition of “no evidence of progression” incorporating complementary measures. The occurrence of worsening within one or more components should then trigger reevaluation of the benefit–risk ratio and consideration of DMT change (see Figure 2).

## 5. Conclusions

Several outcome measures have been used in the phase III clinical trials in PMS, with EDSS being most commonly accepted by regulatory agencies, which is unlikely to change in the near future. However, combining it with other measures to create composite outcome measures may provide better sensitivity to detect disease progression. Therefore, a novel trial methodology and development of sensitive and clinically meaningful outcome measures and biomarkers is urgently needed.

In the hopefully not-too-distant future, more and more potentially neuroprotective drugs are going to be studied. In order to identify effective drugs within reasonable time periods and costs, we need more precise and objective outcome parameters. Paraclinical biomarkers have the potential to detect disease progression earlier and more sensitively, and also reflect the change over time even in patients with stable EDSS. Among those, especially MRI, OCT and sNfL should be included in future phase III clinical trials in order to gain knowledge and facilitate the transfer of these biomarkers to clinical routine. 

With the goal not only to delay, but even to prevent, disease progression, it is time to move beyond clinical outcome measures toward measuring disease progression that is invisible to the naked eye.

## Figures and Tables

**Table 1:** Phase III clinical trials and their designs in progressive multiple sclerosis.

Study Name	Study Design	Inclusion Criteria	Intervention and Comparator	Study Duration	Primary Outcome Measure	Definition of Progression	Sustained Progression	Secondary Outcome Measures
**Primary progressive multiple sclerosis**								
ORATORIO	Randomized, double-blind, placebo-controlled	Age 18–55 years EDSS 3.0–6.5 with pyramidal FS ≥ 2 Duration of MS symptoms of <15 years (EDSS > 5.0) or <10 years (EDSS ≤ 5.0) OCB in the CSF	Ocrelizumab (n = 488) vs. placebo (n = 244)	120 weeks	EDSS	Increase in EDSS score for ≥1.0 point (EDSS ≤ 5.5) or ≥0.5 point (EDSS ≥ 6.0)	12 weeks	24 week CDP, change in T25FW from baseline to week 120 MRI: change in the total volume of brain lesions (T2-weighted) at week 120, change in whole brain volume from week 24 to week 120 PROMs: change in the SF-36 Physical component
INFORMS	Randomized, double-blind, placebo-controlled	Age 25–65 years EDSS 3.5–6.0 with pyramidal FS ≥ 2 Increase in EDSS for ≥0.5 point in the past 2 years T25FW < 30 s Disease duration 2–10 years	Fingolimod (n = 336) vs. placebo (n = 487)	36 months	EDSS-Plus	Increase in EDSS score for ≥1.0 point (EDSS ≤ 5.0) or ≥0.5 point (EDSS ≥ 5.5) or increase of ≥20% in the T25FW or 9HPT	3 months	Time to 3 month CDP MRI: brain volume change, number of Gd-positive lesions, number of T2 lesions PROMs: PRIMUS, EQ-5D, UFIS, MSWS-12
OLYMPUS	Randomized, double-blind, placebo-controlled	Age 18–65 years EDSS 2.0–6.5 with pyramidal FS ≥ 2 OCB in CSF Disease duration ≥1 year	Rituximab (n = 292) vs. placebo (n = 147)	96 weeks	EDSS	Increase in EDSS score for ≥1.0 point (EDSS ≤ 5.5) or ≥0.5 point (EDSS ≥ 6.0)	12 weeks	Time to 24 week CDP, change in MSFC Pharmacodynamic assessments of B-cells counts and immunoglobulin levels MRI: number of Gd-enhancing lesions, change in volume of T2 lesions, change in brain volume
PROMiSE	Randomized, double-blind, placebo-controlled	Age 30–65 years EDSS 3.0–6.5 with pyramidal FS ≥ 2 Evidence of myelopathy for at least 6 months before the screening	Glatiramer acetate (n = 627) vs. placebo (n = 316)	36 months	EDSS	Increase in EDSS score for ≥1.0 point (EDSS ≤ 5.0) or ≥0.5 point (EDSS ≥ 5.5)	3 months	Proportion of progression-free patients, change in EDSS and MSFC MRI: number and volume of T2 lesions, number of Gd-enhancing lesions, brain volume loss
**Secondary progressive multiple sclerosis**								
SPECTRIMS	Randomized, double-blind, placebo-controlled	Age 18–55 years EDSS 3.0–6.5 ≥2 relapses or increase in EDSS for ≥1.0 point in the previous 2 years	Interferon β 1a (n = 360) vs. placebo (n = 358)	36 months	EDSS	Increase in EDSS score for ≥1.0 point (EDSS ≤ 5.5) or ≥0.5 point (EDSS ≥ 6.0)	3 months	Time to becoming wheelchair-bound (EDSS ≥ 7.0), ARR, time to first relapse, proportion of patients with moderate/severe relapses MRI: volume of T2 lesions, number of newly active lesions, change in brain volume
Interferon β 1a study	Randomized, double-blind, placebo-controlled	Age 18–55 years EDSS 1.0–3.5 ≥2 relapses in the prior 3 years No relapse up to 2 months before randomization Disease duration ≥1 year	Interferon β 1a (n = 158) vs. placebo (n = 143)	104 weeks	EDSS	Increase in EDSS score for ≥1.0 point	6 months	Number of relapses MRI: number and volume of Gd-enhancing lesions, T2 lesion volume
IMPACT	Randomized, double-blind, placebo-controlled	Age 18–60 years EDSS 3.5–6.5	Interferon *β* 1a (n = 217) vs. placebo (n = 219)	24 months	MSFC	Decrease in the mean and median MSFC Z-scores	3 months	Change in EDSS, ARR MRI: number of new or enlarging T2 lesions and number of Gd-enhancing lesions PROMs: BDI, MSQLI
Cladribine study	Randomized, double-blind, placebo-controlled	Clinically definite secondary progressive MS for ≥2 years	Cladribine (n = 24) vs. placebo (n = 24)	2 years	EDSS, SNRS	The change in both measurements was calculated every month^1^		MRI: number and volume of T2-lesions CSF: total protein and immunoglobulin concentration, oligoclonal bands
MIMS	Randomized, double-blind, placebo-controlled	Age 18–55 years EDSS 3.0–6.0 Increase in EDSS for ≥1.0 point during 18 months before enrolment (progressive relapsing-remitting or secondary progressive MS) No relapse up to 8 weeks before randomization	Mitoxantrone (n = 124) vs. placebo (n = 64)	24 months	Composite score^2^	Only a change between baseline and 24 months in each measurement of the composite score was calculated		Proportion of patients with deterioration of ≥1.0 EDSS point, proportion of patients with such deterioration confirmed after 3 and 6 months, time to first EDSS deterioration, time to first relapse, ARR, proportion of patients without relapse, number of days in hospital, use of wheelchair assistance MRI: number and volume of Gd-enhancing, and nonenhancing T1 and T2 lesions PROMs: quality of life (Stanford health assessment questionnaire)
ASCEND	Randomized, double-blind, placebo-controlled	Age 18–58 years EDSS 3.0–6.5 with disability progression during the year before enrolment MSSS ≥ 4 No relapse up to 3 months before randomization	Natalizumab (n = 439) vs. placebo (n = 448)	96 weeks	EDSS-Plus	Increase in EDSS score for ≥1.0 point (EDSS ≤ 5.5) or ≥0.5 point (EDSS ≥ 6.0) or increase of ≥20% in the T25FW or 9HPT	3 months	Proportion of patients with improvement in T25FW, proportion of patients with disability progression, measured by individual physical EDSS FS scores MRI: change in whole brain volume between week 24 and 96 PROMs: change in patient-reported ambulatory status (MSWS-12), manual ability (ABILHAND questionnaire), quality of life (MSIS-29 physical score)^3^
EXPAND	Randomized, double-blind, placebo-controlled	Age 18–60 years EDSS 3.0–6.5 progression during 2 years before enrolment No relapse up to 3 months before randomization	Siponimod (n = 1105) vs. placebo (n = 546)	Protocol amended after occurrence of 374 CDP events and after ≥95% of patients had been assigned to treatment for ≥12 months	EDSS	Increase in EDSS score for ≥1.0 points (EDSS ≤ 5.0) or increase of ≥0.5 (EDSS ≥ 5.5)	3 months	Time to 3 and 6 month confirmed worsening of T25FW, ARR, time to first relapse, proportion of relapse-free patients MRI: number of new or enlarging T2 lesions, change in T2 lesion volume, number of Gd-enhancing lesions, brain volume change PROMs: change in score on the MSWS-12

^1^ Meaningful change was defined as an increase in EDSS for ≥1.0 point or decrease in SNRS for ≥10 points after one year. ^2^ Composite score of EDSS, ambulation index, number of treated relapses, time to first treated relapse, and standardized neurological status. ^3^ Extended trial included additional secondary endpoints: proportion of patients with disability progression on the multicomponent endpoint during additional follow-up time, change in EDSS, T25FW, and 9HPT from baseline to week 156, change in whole brain and grey matter volume, change in number of T2 lesions, change in 6-Minute Walk Test, MSIS-29 Physical, and SDMT, and change in Work Productivity and Activity Impairment Questionnaire. ARR: annualized relapse rate, BDI: Beck Depression Inventory, CDP: confirmed disease progression, CSF: cerebrospinal fluid, EDSS: Expanded Disability Status Scale, EQ-5D: EuroQoL, MRI: magnetic resonance imaging, MS: multiple sclerosis, MSFC: Multiple Sclerosis Functional Composite, MSIS-29: Multiple Sclerosis Impact Scale-29, MSWS-12: 12-item Multiple Sclerosis Walking Scale, OCB: oligoclonal bands, PRIMUS: Patient-Reported Indices in Multiple Sclerosis, PROMs: patient-reported outcome measures, QoL: quality of life, SDMT: Symbol Digit Modalities Test, SF-36: Medical Outcomes Study 36-Item Short-Form Health Survey, SNRS: Scripps neurologic rating score, T25FW: Timed 25-Foot Walk, UFIS: Unidimensional Fatigue Impact Scale, 9HPT: 9-Hole Peg Test.

## Data Availability

No new data were created or analyzed in this study. Data sharing is not applicable to this article.
